# Pediatric Anesthetic Management of a Patient With an ALG‐13 Gene Mutation, a Rare Congenital Disorder of Glycosylation

**DOI:** 10.1002/ccr3.70027

**Published:** 2025-01-16

**Authors:** Esha Thakkar, John A. Iasiello, Sunny R. Cai, Adrienne Hutton

**Affiliations:** ^1^ Brody School of Medicine at East Carolina University Greenville North Carolina USA

**Keywords:** ALG‐13 mutation, congenital disorders of glycosylation, malignant hyperthermia, pediatric anesthesia, pharmacology

## Abstract

Congenital disorders of glycosylation are rare and present a challenge in management due to interactions with intraoperative medications. We present safe and successful anesthetic management of a pediatric patient with an ALG‐13 gene mutation.

## 1 Introduction

Congenital disorders of glycosylation are rare disorders with few case reports published in the literature. Patients can present with many different symptoms such as microcephaly, developmental delay, and hypotonia [1]. The few case reports published in the literature offer medications that were safely used in pediatric patients. This case report adds to the literature by providing insight into management of a patient with an ALG‐13 gene mutation.

## 2 Case Report

### 2.1 Case History/Examination

A 3‐year‐old male with recurrent, acute bilateral otitis media presented for an adenoidectomy and bilateral myringotomy. Past medical history was significant for an ALG‐13 gene mutation, seizures, and global developmental delay with no known cardiac or hepatic issues. Family history was significant for malignant hyperthermia (MH) in a paternal uncle.

## 3 Methods

Secondary to a family history of MH, the decision was made to avoid triggering volatile anesthetics such as isoflurane, sevoflurane, and desflurane. The anesthesia machine was flushed per manufacture protocol with high‐flow oxygen and charcoal filters [2]. Preoperative management involved oral midazolam (0.5 mg/kg for a total of 8.5 mg) and inhaled nitrous oxide to achieve mild sedation and anxiolysis for IV placement combined with distraction therapy and light restraint of extremity by staff. Intravenous 15 mcg fentanyl, 1 mg midazolam, and inhaled nitrous were used for induction; the patient was briefly bradycardic to a heart rate of 61 but resolved spontaneously following intubation (5 min from induction start). Twenty milligrams of rocuronium was used for neuromuscular blockade and intubation was performed successfully on the first attempt under direct visualization with a 3.5 mm cuffed endotracheal tube and a miller 1.5 blade. For maintenance anesthesia, nitrous oxide and a remifentanil infusion at 0.1 mcg/kg/min was administered. For neuromuscular reversal, four twitches were present and 30 mg sugammadex was administered. The patient tolerated the procedure without difficulty and was discharged on the same day as the procedure.

## 4 Discussion

Congenital disorders of glycosylation (CDG) comprise over 130 rare metabolic disorders via mutations in specific genes that often impact multiple organ systems. The process of glycosylation occurs when a carbohydrate is attached to a protein or lipid, leading to the formation of glycoproteins and glycolipids, respectively. Both glycoproteins and glycolipids have important functions in all tissues and organs and a deficiency can lead to consequences throughout the body [3]. Specifically, the mutation in the gene ALG‐13 affects N‐linked glycosylation which can disrupt various metabolic pathways and often manifests as microcephaly, hepatomegaly, seizures, developmental delay, and generalized hypotonia [1]. The incidence and prevalence of CDG is less than one in 1,000,000 [4] and spans worldwide to almost every ethnic background and affects both sexes equally [5]. Most CDG conditions follow an autosomal recessive inheritance, but the large variety can include autosomal dominant or X‐linked inheritance [3]. ALG‐13, located on the X‐chromosome, is typically a new genetic mutation or follows an X‐linked recessive inheritance [6]. To date, there are no specific guidelines for anesthetic management in CDG patients and there are few case reports outlining optimal or successful management. This case demonstrates a safe and successful anesthetic in a patient with CDG while exemplifying the importance of taking all aspects of a patient's presentation and history into consideration when identifying an optimal anesthetic plan.

Due to a positive family history of MH, triggering agents such as volatile anesthetics and succinylcholine were avoided for management [7]. To adequately purge the anesthesia machine of volatile anesthetics, the machine was flushed as specified above. While volatile anesthetics such as sevoflurane and isoflurane can trigger MH, nitrous oxide is not a triggering agent for MH. Often a total intravenous anesthetic (TIVA) is utilized to avoid MH triggers [8]; however, for a pediatric patient, nitrous oxide is a helpful sedative which can be administered via inhalation while intravenous access is yet to be obtained [9, 10]. Additionally, there is no known interference between nitrous oxide and the glycosylation process.

While there are no known contraindications for succinylcholine use in patients with CDG, it is a triggering agent for MH. Depolarizing neuromuscular blocking agents such as succinylcholine can have a prolonged effect in patients with hypotonia and CDG patient susceptibility to nondepolarizing neuromuscular blocking agents has not been defined but these agents have been used [10, 11]. Rocuronium, a nondepolarizing neuromuscular blocker, and sugammadex, a potent binding reversal agent, were used for neuromuscular blockade and reversal, respectively [10, 12]. Propofol can interfere in glycosylation pathways and studies caution against its use in CDG patients due to potential adverse effects, thus we opted to avoid propofol throughout the anesthetic management [13].

**FIGURE 1:**
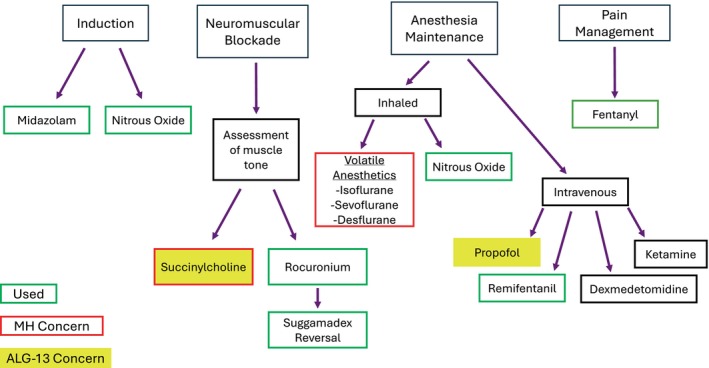
Anesthesia management considerations for the ALG‐13 patient.

Two other agents considered were ketamine and dexmedetomidine. Both ketamine and dexmedetomidine have an unknown impact on the glycosylation process. Ketamine has been considered controversial in patients with a history of seizures or epilepsy, although studies do not support avoidance. We kept ketamine in mind as an alternative agent. Dexmedetomidine, as an alpha‐2 agonist, can cause bradycardia due to its sympatholytic effect [14]. Since the pediatric patient was bradycardic on induction, dexmedetomidine was avoided. Remifentanil infusion has been shown to reduce postoperative nausea and vomiting and provide faster recovery in patients with CDG and was used successfully in this patient [12]. For pain management, fentanyl was used; acetaminophen was considered but was not used due to potentially harmful impact on hepatic function, as there has been a high prevalence of hepatic dysfunction in CDG patients [10, 15, 16]. Figure 1 shows a flowchart on management of this patient's condition along with alternative anesthetic considerations.

Due to the development of craniofacial abnormalities, CDG patients can present with a potentially difficult airway and must be evaluated. The definition of a difficult airway by the Pediatric Difficult Intubation Registry includes (1) failure to visualize vocal cords on direct laryngoscopy (DL) by an experienced provider, (2) impossible DL due to abnormal anatomy, (3) failed DL within the last 6 months, or (4) DL felt to be harmful in a patient with suspected difficult laryngoscopy [17]. A primary anatomic concern for CDG is the development of microcephaly which raises concerns for difficult laryngoscopy due to a narrow mouth opening, limited mobility of the mandible, and small palate. Options outside of direct laryngoscopy include video laryngoscopy, supraglottic airway, and flexible fiberoptic bronchoscope and optical stylet with the fiberoptic bronchoscope currently considered the gold standard for difficult airway management in pediatric patients [17]. In the event of inability to intubate and inability to ventilate in a pediatric patient, surgical access through the cricothyroid membrane or anterior tracheal wall is an emergent option. The surgeon, an otolaryngologist, was present for induction during our case, and it is important to have a physician able to perform surgical access present throughout induction and peri‐intubation for any pediatric patient considered a possible difficult airway.

## 5 Conclusion and Results

Overall, this case report demonstrated successful anesthetic management for a pediatric patient with a congenital disorder of glycosylation and a family history of malignant hyperthermia. Congenital disorders, such as ALG‐13, can impact anesthetic management not only by medication and anesthetic interactions but also via anatomic phenotypes which can increase morbidity and mortality to the presence of a difficult airway [15]. We highlight the importance of a comprehensive evaluation to factor in all anesthetic considerations.

## Author Contributions

**Esha Thakkar:** writing – original draft. **John A. Iasiello:** data curation, investigation. **Sunny R. Cai:** formal analysis, writing – review and editing. **Adrienne Hutton:** data curation, investigation.

## Consent

Written informed consent was obtained from the patient to publish this case report.

## Data Availability

Data sharing is not applicable to this article as no new data were created or analyzed in this study.

## References

[1] “ALG13‐CDG ‐ About the Disease ‐ Genetic and Rare Diseases Information Center,” rarediseases.info.nih.gov, https://rarediseases.info.nih.gov/diseases/12401/alg13‐cdg Orphanet: ALG13‐CDG, www.orpha.net, https://www.orpha.net/en/disease/detail/324422.

[2] L. H. Schönell , C. Sims , and M. Bulsara , “Preparing a New Generation Anaesthetic Machine for Patients Susceptible to Malignant Hyperthermia,” Anaesthesia and Intensive Care 31, no. 1 (2003): 58–62, 10.1177/0310057X0303100112. 12635397

[3] “Congenital Disorders of Glycosylation ‐ Symptoms, Causes, Treatment: Nord,” National Organization for Rare Disorders, January 12, 2023, https://rarediseases.org/rare‐diseases/congenital‐disorders‐of‐glycosylation/.

[4] I. J. Chang , M. He , and C. T. Lam , “Congenital Disorders of Glycosylation,” Annals of Translational Medicine 6, no. 24 (2018): 477, 10.21037/atm.2018.10.45. 30740408 PMC6331365

[5] T. Cai , J. Huang , X. Ma , et al., “Case Report: Identification of Two Variants of ALG13 in Families With or Without Seizure and Binocular Strabismus: Phenotypic Spectrum Analysis,” Frontiers in Genetics 13 (2022): 892940, 10.3389/fgene.2022.892940. 35899201 PMC9310169

[6] H. Rosenberg , M. Davis , D. James , N. Pollock , and K. Stowell , “Malignant Hyperthermia,” Orphanet Journal of Rare Diseases 2 (2007): 21, 10.1186/1750-1172-2-21. 17456235 PMC1867813

[7] P. K. Gupta , J. G. Bilmen , and P. M. Hopkins , “Anaesthetic Management of a Known or Suspected Malignant Hyperthermia Susceptible Patient,” BJA Education 21, no. 6 (2021): 218–224, 10.1016/j.bjae.2021.01.003. 34026275 PMC8134759

[8] N. J. Halliday , “Malignant Hyperthermia,” Journal of Craniofacial Surgery 14, no. 5 (2003): 800–802, 10.1097/00001665-200309000-00039. 14501352

[9] W. Sakai , Y. Yoshikawa , Y. Tokinaga , and M. Yamakage , “Anesthetic Management of a Child With Phosphomannomutase‐2 Congenital Disorder of Glycosylation (PMM2‐CDG),” JA Clinical Reports 3, no. 1 (2017): 8, 10.1186/s40981-017-0080-y. 29492447 PMC5813674

[10] E. C. Chang , Y. H. Chang , Y. S. Tsai , Y. L. Hung , M. J. Li , and C. S. Wong , “Case Report: The Art of Anesthesiology‐Approaching a Minor Procedure in a Child With MPI‐CDG,” Frontiers in Pharmacology 13 (2022): 1038090, 10.3389/fphar.2022.1038090. 36588700 PMC9798425

[11] T. Voss , A. Wang , M. DeAngelis , et al., “Sugammadex for Reversal of Neuromuscular Blockade in Pediatric Patients: Results From a Phase IV Randomized Study,” Pediatric Anesthesia 32, no. 3 (2021): 436–445, 10.1111/pan.14370. 34878707

[12] A. Lehavi , H. Mandel , and Y. Katz , “Anesthetic Management of a Boy With Congenital Disorder of Glycosylation (CDG) I‐x,” International Journal of Clinical Medicine 2, no. 3 (2011): 325–327, 10.4236/ijcm.2011.23056.

[13] H. Lei , L. Chao , T. Miao , et al., “Incidence and Risk Factors of Bradycardia in Pediatric Patients Undergoing Intranasal Dexmedetomidine Sedation,” Acta Anaesthesiologica Scandinavica 64, no. 4 (2020): 464–471, 10.1111/aas.13509. 31736052

[14] D. Marques‐da‐Silva , F. V. Dos Reis , M. Monticelli , et al., “Liver Involvement in Congenital Disorders of Glycosylation (CDG). A Systematic Review of the Literature,” Journal of Inherited Metabolic Disease 40, no. 2 (2017): 195–207, 10.1007/s10545-016-0012-4. 28108845

[15] E. Schollen , C. G. Frank , L. Keldermans , et al., “Clinical and Molecular Features of Three Patients With Congenital Disorders of Glycosylation Type Ih (CDG‐Ih) (ALG8 Deficiency),” Journal of Medical Genetics 41, no. 7 (2004): 550–556, 10.1136/jmg.2003.016923. 15235028 PMC1735831

[16] A. S. Huang , J. Hajduk , C. Rim , S. Coffield , and N. Jagannathan , “Focused Review on Management of the Difficult Paediatric Airway,” Indian Journal of Anaesthesia 63, no. 6 (2019): 428–436, 10.4103/ija.IJA_250_19. 31263293 PMC6573050

[17] J. E. Fiadjoe , A. Nishisaki , N. Jagannathan , et al., “Airway Management Complications in Children With Difficult Tracheal Intubation From the Pediatric Difficult Intubation (PeDI) Registry: A Prospective Cohort Analysis,” Lancet Respiratory Medicine 4, no. 1 (2016): 37–48, 10.1016/S2213-2600(15)00508-1. 26705976

